# The development and education of a workforce in childhood cancer services in low- and middle-income countries: a scoping review protocol

**DOI:** 10.1186/s13643-022-02040-0

**Published:** 2022-08-13

**Authors:** Julie Cayrol, André Ilbawi, Michael Sullivan, Amy Gray

**Affiliations:** 1 The University of Melbourne, Melbourne, Australia; 2 Murdoch Children’s Research Institute, Melbourne, Australia; 3 World Health Organization, Geneva, Switzerland; 4 The Royal Children’s Hospital, Melbourne, Australia

**Keywords:** Workforce, Health professionals, Human resources, Childhood cancer, Paediatric oncology, Training, Health systems

## Abstract

**Background:**

An estimated 400,000 children develop cancer worldwide. Of those, 90% occur in low- and middle-income countries, where survival rates can be as low as 30%. To reduce the childhood cancer survival gap between high- and low- and middle-income countries (LMIC), the World Health Organization launched the Global Initiative for Childhood Cancer in 2018, to support governments in building sustainable childhood cancer programs, with the aim to increase access and quality of care for children with cancer. Developing a high-quality and trained workforce is key to the success of childhood cancer services, but more information is needed on the interventions used to develop and train a workforce. The objective of this review is to understand the key factors described in the literature in relation to the development and training of a workforce in childhood cancer (defined here as ages 0–19) in LMIC, including challenges, interventions and their outcomes.

**Methods:**

We will include sources of evidence that describe the development or training of a childhood cancer workforce in health services that diagnose, refer or treat children and adolescents with cancer, in low- and middle-income countries as defined by the World Bank. The following databases will be searched: OVID Medline, Embase and Pubmed from 2001 to present with no restriction of language. Grey literature searches will also be performed in Proquest Dissertation and Theses, as well as relevant organizations’ websites, and conference proceedings will be searched in conference websites. In addition, references lists will be reviewed manually. Two people will screen abstracts and full-texts and extract data. Data will be presented in a table or chart, with an accompanying narrative summary responding to the review questions. A framework synthesis will be conducted: data will be charted against a framework adapted from the 2016 WHO Global Strategy for Human Resources for Heath: Workforce 2030.

**Discussion:**

This scoping review will allow to map the existing literature on workforce development in LMIC, identify potential interventions and highlight data and knowledge gaps. This constitutes a first step towards adopting successful strategies more broadly, formulating research priorities and developing effective policies and interventions.

**Systematic review registration:**

Open Science Framework osf.io/3mp7n

**Supplementary Information:**

The online version contains supplementary material available at 10.1186/s13643-022-02040-0.

## Background

With increasing life expectancy and epidemiological and demographic transitions of settings previously burdened by infectious diseases, the burden of cancer is rising worldwide [1–3]. Childhood and adolescence cancers contribute significantly to the global cancer burden. It is estimated that 400,000 children aged 0–14 years developed cancer worldwide in the year 2015. However, of these children, only half received a diagnosis, leaving approximately 200.000 children undiagnosed [4]. Of children who develop cancer, approximately 90% occur in low- and middle- income countries (LMIC, as defined by the World Bank country classifications), settings in which nearly 95% of the world’s children live. In addition, a large proportion of undiagnosed cancer cases occur in regions containing LMIC, such as Eastern and Western Africa, South-East Asia and South-Central Asia [4].

The likelihood of a child with cancer surviving depends greatly on the health system context of the country in which they live and on the socioeconomic status of the child’s family. Survival rates are over 80% in high-income countries (HIC), but less than 30% in lower-middle-income and low-income countries (LIC) [5–8]. In addition, survival rates have stayed stable in LMIC, where the medical progress achieved in HIC has not been translated.

In low-resourced settings, the most common reasons for inferior survival are delayed or missed diagnoses due to lack of expertise or inadequate referral systems, lack of diagnostic and treatment capacity, poor treatment quality or lack of supportive care, treatment abandonment due to high costs, inadequate access to services or misconceptions from parents and caregivers [9].

To address these inequalities, the World Health Organization (WHO) launched the Global Initiative for Childhood Cancer (GICC) in 2018, aiming to improve access to and quality of care for children with cancer. Its goal is to “increase the overall survival of children with cancer to 60% by 2030, while reducing suffering for all children” (Page 34, World Health Organization. WHO Global Initiative for Childhood Cancer: Technical package [Internet] 2021) [9]. The GICC seeks to support governments in building sustainable childhood cancer programs. The Cure*All* framework, conceptualized by the WHO and based on four pillars and three enablers, is a guide for countries implementing the GICC. One of the core pillars of the Cure*All* Framework is to “create centres of excellence with sufficient and competent workforce to deliver high quality care” (Page 43, World Health Organization. WHO Global Initiative for Childhood Cancer: Technical package [Internet] 2021).

In LMIC, the effective mobilisation of a workforce is key to strengthening health systems and improving health outcomes [10]. Unfortunately, the global health workforce shortage, due to the lack of trained or employed health workers, particularly affects LMIC and contributes to poorer health outcomes [11]. This not only affects primary health workers but also specialists such as the ones needed for the diagnosis and management of childhood cancer [12]. To combat this issue, international agencies have taken action: the 2006 World Health Assembly urged Member States to scale-up the production of health workers, by increasing training and education, along with the development of strategies to optimize health workforce and planning [13]. In 2014, the World Health Assembly resolution included ongoing commitments towards the development of Human Resources for Health to achieve Universal Health Coverage [14]. The over-arching objective is to align the health workforce with the health needs of the population, by training and expanding a workforce of sufficient quality, quantity and relevance, in order to improve population health outcomes [11].

To reduce the survival gap between HIC and LMIC, evolving childhood cancer services are being developed worldwide. As a part of this, developing a high-quality and trained workforce is key to the success of childhood cancer programs, to ensure accurate and timely diagnoses, curative and supportive care treatments of high quality and ongoing survivorship care.

Preliminary literature searches reveal different methods for capacity building, often involving partnerships with other institutions. These including tele-mentoring programs, long-term twinning partnerships and regional schools for training of health professionals [15–18]. In French-speaking Africa, an African School of Paediatric Oncology (EAOP) was established in 2012 as a training program supported by a Non-Governmental Organization (NGO), the Sanofi Espoir Foundation. Since then, the pool of qualified paediatric oncology professionals has increased [16]. Some studies focus on describing the perspective of healthcare workers in this field of medicine. In Indonesia, interviews to healthcare providers working in a childhood cancer service showed that providers communicate less effectively and offer less support to families of low resources, potentially affecting their adherence to treatment [19]. In relation to nursing care, in response to the absence of specialised education and the unsafe work environments that nurses are exposed to [20], baseline standards for the provision of safe and effective nursing care in LMIC have been published by the SIOP (International Society for Paediatric Oncology) PODC (Paediatric Oncology in Developing Countries) Nursing Working Group [21].

Mapping and synthesizing the literature on the childhood cancer workforce (its challenges, the interventions adopted to develop it, as well as their outcomes) would be helpful for researchers and policy-makers implementing childhood cancer services. This would allow to identify challenges, data gaps and potential interventions which could lead to new policy, further research or broader adoption of successful strategies.

A preliminary search for existing scoping reviews and systematic reviews on this topic was conducted in Pubmed, PROSPERO, Cochrane database of systematic reviews, JBI Database of Systematic Reviews and Implementation Reports and Cumulative Index to Nursing and Allied Health Literature (CINAH) on 19 July 2021. Two systematic reviews are ongoing and connected to our review. However, both reviews have different aims and inclusion criteria. An ongoing systematic review is examining the current cancer workforce capacity across adult and childhood cancer care (including cancer occupation ratios, the existence of cancer workforce plans, and the distribution and definition of occupations) in countries of all income levels (“Current cancer workforce capacity: systematic review of cancer occupation ratios”, Ilbawi et al., registered in PROSPERO ID CRD42018095414). The purpose of the proposed scoping review is not to document ratios or specific occupation definitions. A second systematic review is analysing the outcomes of strategies for workforce capacity building and scale-up for adult cancers (with a focus on breast and cervical cancers), in all geographical regions (not limited to LMIC) (“Human resources for comprehensive cancer care: a systematic review of strategies for cancer workforce capacity building and scaling- up”. Trapani, Ilbawi, PROSPERO ID: CRD42020109377). While we appreciate some of the strategies identified in the second systematic review may pertain or be applicable to the childhood cancer context, no review has been undertaken to specifically map the literature and summarise findings related uniquely to the childhood cancer workforce and limited to LMIC.

The objective of this scoping review is to understand the key factors described in the literature in relation to workforce development and training in childhood cancer in LMIC. This includes summarising and cataloguing findings on this topic and identifying gaps and opportunities for developing future research and policy guiding the implementation of childhood cancer services in LMIC. This protocol was registered on September 9, 2021, in Open Science Framework (osf.io/3mp7n).

## Methods/design

### Study design

Systematic reviews seek to appraise and synthesise research evidence and are commonly used to assess the effectiveness of health interventions [22]. They aim to respond to a narrow question, where there is knowledge that an appropriate and often small number of quality assessed studies are available [23]. In contrast, the scoping review methodology seeks to identify the nature and extent of the literature—to understand what is available, regardless of study design, in disciplines in which evidence is emerging and there is an expected scarcity of specific study designs such as randomized controlled trials [24]. The scoping review methodology allows to capture all types of evidence (including grey literature), which is suited to topics which have had less formal scholarship. Scoping reviews respond to broader review questions and by mapping the literature, help identify gaps in research and data [25, 26], while being subject to the same methodological rigour as systematic reviews. The scoping review methodology is appropriate for the proposed review, given the lack of research in these topic areas and the unlikelihood of finding the type of evidence that would be most appropriate for a systematic review, such as randomized controlled trials.

The proposed scoping review will be conducted in accordance with the JBI methodology for scoping reviews [26, 27], which was updated in 2021 from its original version published in 2015, providing guidance for authors of scoping reviews to rigorously conduct their reviews. The proposed scoping review will be reported in accordance with the Preferred Reporting Items for Systematic Reviews and Meta-Analyses extension for Scoping Reviews (PRISMA-ScR), and a checklist will be provided in the final review.

### Review questions

This scoping review aims to respond to the following main question and sub-questions: “What is known in the literature on the development and training of a childhood cancer workforce in low- and middle-income countries?”

Sub-questions include:

- “What challenges have been described for the childhood cancer workforce in LMIC?”
- “To which professions (e.g.: physicians, nursing, psycho-oncologists) and to which income levels (within LMIC) does the literature relate?”
- “What strategies or interventions have been described to develop, train and sustain the workforce?”
- “What are the outcomes of these interventions, and how are they measured?”

### Inclusion criteria

The inclusion criteria will follow the PCC (Population, Concept, Context) mnemonic as proposed by the JBI methodology.

### Participants

The review will consider studies that include health professionals involved in the diagnosis and management of children and adolescents with cancer (defined here as aged 0–19) across the cancer continuum, from early detection and referral in primary care settings, to diagnosis, treatment, palliative care and survivorship. Cancer is defined by the National Cancer Institute as “a disease in which some of the body cells grow uncontrollably and spread to other parts of the body”. This review will however include services that diagnose and treat both benign and malignant neoplasms, given these conditions are often treated in the same centres, by the same health professionals as part of a multi-disciplinary team. The health professionals may include physicians, surgeons, radiation oncologists, anatomical pathologists, radiation therapists, pharmacists, nursing staff, allied health staff, psycho-oncologists, patient navigators and palliative care staff. This review will also include health professionals working in primary care as well as in secondary and tertiary cancer services. Understanding that the childhood cancer workforce is composed of a large, multi-disciplinary team that is interdependent, we have included a broad variety of professions in the inclusion criteria.

### Concept

This review will consider studies that explore the development and training of a childhood cancer workforce.

Included sources will be those that focus on or mention the workforce in childhood and adolescence cancer services even if this is not the primary focus of the document. This includes literature that describes the current capacity and challenges for health professionals in these emerging services; their perceptions; the interventions used to develop and educate the workforce, and if available, the outcomes of these interventions. Including described challenges will allow identification of areas in which future interventions could take place.

Excluded sources will be those that refer only to adult cancer services, or to service delivery challenges, program development or results, with no specific mention of the workforce. Articles referring to the development of palliative care services will only be included in the context of childhood cancer palliative treatment and when workforce is addressed.

### Context

This review will consider studies conducted in health services that diagnose, refer or treat children with cancer in low- and middle-income countries (as defined by the World Bank), such as primary care centres, public and private hospitals. Articles which focus on mixed adult/child and adolescent cancer services will also be included, given this is a frequent model of care in LMIC.

We recognise that access and quality of health care systems vary greatly between countries of the same income group. As such, childhood cancer services can be heterogeneous even in countries belonging to the same income group or geographical region. However, World Bank income groups are commonly used in the scientific literature and WHO publications to describe country settings as an umbrella term for low-resourced and resource-limited settings and therefore will be used in this review protocol to define the population of interest. In addition, we anticipate some of the challenges and interventions to be similar in different countries and therefore have chosen the broader LMIC context for this study. We expect the amount of literature to be particularly scarce from low-income countries, and therefore, we will include all middle- (upper-middle and lower-middle) and low-income countries. Articles and documents referring to high income countries only will be excluded.

It is anticipated that the inclusion and exclusion criteria may be refined in an iterative process as the research team becomes more familiar with the available literature.

### Types of sources

This scoping review will consider quantitative, qualitative and mixed methods study designs for inclusion. In addition, review articles, conference abstracts, comments, letters, editorials, opinion papers and grey literature such as theses, web material, Government and non-Governmental documents such as country reports, policies and regulations will also be considered for inclusion. Case reports, clinical guidelines and practice guidelines will be excluded given they are not relevant to the question.

This being an emerging field, in which primary research studies are rare, we expect that a diversity of literature may be present in conference proceedings and grey literature from country reports and policies.

### Search strategy

The search strategy will aim to locate both published and unpublished primary studies, reviews, abstracts, comments, letters, editorials and opinion papers.

A preliminary search was conducted in Ovid Medline to identify articles on the topic. The text words contained in the titles and abstracts of relevant articles, and the index terms used to describe the articles were used to develop a full search strategy for Ovid Medline with the help of a medical librarian at The Royal Children’s Hospital. (see Supplementary file 1). The search was refined with other members of the team (AI, AG). The search strategy, including all identified keywords and index terms, was adapted for each included database.

Given how broad the topic is, the search strategy combines two searches. The first search focuses on post-graduate education and training of the workforce, while the second search explores workforce issues more broadly (such as planning, development, workforce as part of a health systems building block). Both of these searches are combined with Specific Medical Subjects Headings (MeSH) and key words related to “children” and “cancer”. A low- and middle-income country filter developed by the Medical Library at The Royal Children’s Hospital was applied and combined with the previous search terms. The list of countries was verified against historical World Bank data and includes all countries at any time classified by World Bank as LMIC, since 1990. The two searches have then been combined in the search engines given we expect there may be some duplication of findings. The search will span the last 20 years, from January 1, 2001, to present (although we anticipate that most of the literature will be from the last 10 years given this is the period during which more attention was brought to the burden of childhood cancer in LMIC). No language limit will be applied, however the search itself will be performed in English.

We expect that the search strategy may be iterative as we become more familiar with the evidence, keywords and sources. In this case, the search will be tracked in a search logbook and transparently reflected in the final review.

The databases to be searched are Ovid Medline, Ovid Embase and Pubmed. Cinahl and Web of science will be searched to capture nursing and allied health literature. We anticipate these will be sufficient to capture the peer-reviewed literature.

Grey literature searches will also be undertaken, in Proquest Dissertation and Theses, as well as relevant organizations’ websites that relate to childhood cancer and global health. Examples of these include the following: WHO website, WHO Global Index Medicus for all regions, International Agency for Research on Cancer (IARC), World Bank website, https://www.who.int/workforcealliance/en/, Human resources for Health https://www.hrhresourcecenter.org/, www.capacityproject.org. Websites of the following key charitable organisations will also be searched: American Childhood Cancer Organisation, Canadian Cancer Society, Children’s Cancer and Leukaemia Group (CCLG) and Childhood Cancer International. Other websites will be determined via snowball effect as the search progresses.

Additionally, the references lists of articles included in the review will be checked manually and screened for additional papers. Conference proceedings will be identified in the database searches and screened the same way as full text articles.

### Study selection

Following the search, all identified records will be collated and uploaded into Endnote X8 (Clarivate Analytics, PA, USA) and subsequently to Covidence (Veritas Health Innovation, Melbourne, Australia). Duplicates will be removed in Covidence. Titles and abstracts will be screened independently in Covidence by two reviewers reviewer (JC and AG or MS) based on the inclusion criteria to exclude any obvious exclusions. At the start of the screening, the inclusion criteria will be refined amongst the group to resolve any issues. Where there is disagreement, a third reviewer will resolve any conflict.

Articles in a language other than English will either be assessed by a reviewer fluent in that language (JC: Spanish, French). For languages other than those three languages, abstracts will be screened requesting translational help via inter-departmental emails within the different institutions to which the authors are affiliated to. If the article reaches full text screening or data extraction phase, this will be performed by a staff member fluent in this language in exchange for acknowledgement in the publication. The full texts of selected citations will be assessed in detail against the inclusion criteria by two independent reviewers. Reasons for exclusion of full-text papers that do not meet the inclusion criteria will be recorded in a logbook and reported in the scoping review. Any disagreements will be resolved through discussion or with a third reviewer. If additional citations were to be identified from the manual review of the references, or if the search strategy were to change, these would be added and undergo the same process. The results of the search will be reported in full in the final scoping review and presented in a Preferred Reporting Items for Systematic Reviews and Meta-analyses for Scoping Reviews (PRISMA-ScR) flow diagram [28]. According the scoping review methodology, a critical appraisal of the quality of studies will not be undertaken.

### Data extraction

Data will be extracted from papers using a data extraction tool developed by the reviewers. The data extracted will include specific details about the source (author, year of publication, reference), main document objective, methodology, country of origin and context (income level, health service context), participants and findings relevant to the research question (type of challenge described, nature and duration of intervention, outcome of intervention). The data extraction table has been adapted from the JBI template data extraction instrument [27] and a draft extraction tool is provided (Supplementary file 2). The use of the data extraction tool will be piloted by two members of the team for the first 5 sources of evidence. The draft data extraction tool will be modified and revised as necessary during the process of extracting data and modifications will be detailed in the full scoping review. Data will be extracted by one reviewer (JC) and checked by another reviewer. Any disagreements that arise will be resolved through discussion or with a third reviewer. Authors of papers will be contacted to request missing or additional data, where required.

### Data analysis and presentation

Data will be collated and results will be synthesised according to the type of source, context and key themes using descriptive qualitative content analysis [25], though a thematic qualitative analysis will not be performed as this is not the aim of a scoping review [26]. Data will be presented per geographical region and income level, and per professions described in the literature, to respond to the review sub-questions. These data will be presented with basic numerical analysis (counts and percentages of studies per income level and geographical region, and per type of profession). Data will be mapped with a table and organised to present the types of challenges and interventions described in scaling-up a childhood cancer workforce in LMIC, as well as their outcomes, and how outcomes are measured and influenced. A narrative summary will accompany the tabulated and/or charted results and will describe how the results relate to the review’s questions.

Finally, a framework synthesis will be conducted: data will be sorted and charted against a framework adapted from the 2016 Global Strategy for Human Resources for Health: Workforce 2030 and the Human Resources for Health Action framework. The Global Strategy: Workforce 2030 document, published by the WHO Workforce Department in consultation with the Global Health Workforce Alliance and worldwide health workforce experts, is the most recent guidance document aimed at policy-makers needing to respond to health workforce challenges. This framework has the goal to ensure availability, accessibility, acceptability, coverage and quality of health workers, with the aim to attain Universal Health Coverage and strengthen health systems [29]. It is based on 4 main actions that include several strategies. The Human Resources for Health Action framework [30], published in 2009, was developed by the Global Health Workforce Alliance as a collaboration between USAID and the WHO. It includes six action fields (policy, finance, education, partnership, leadership and human resources management systems), implemented across 4 phases (situational analysis, planning, implementation, monitoring and evaluation).

Data will be mapped against this adapted framework to identify areas of workforce development in which interventions have taken place and their outcomes. Finally, mapping the data against the framework will help identify and report gaps in the literature and future research priorities.

### Stakeholder consultation

Stakeholder consultations are an optional step in scoping reviews as per the JBI methodology, although has been considered by some as a necessary one to increase the validity of the review [24]. We will not perform a stakeholder consultation as part of this review given this will be conducted as a separate qualitative project aiming to understand the strategies to develop a childhood cancer workforce in low-resourced settings.

## Discussion

Developing a competent workforce is an essential component of improving access and quality of care for children and adolescents with cancer in LMIC. This study is part of a larger project with the aim to understand the key factors related to the development of a childhood cancer workforce in LMIC, from a training and health systems perspective.

This review will collate and summarise literature on the training and development of a childhood cancer workforce in LMIC. By mapping the literature and determining knowledge gaps, this scoping review will constitute a first step towards identifying research priorities and developing effective policies and interventions for countries. While this review will follow rigorous methodology, we anticipate some limitations. Being a scoping review, an appraisal of quality of studies will not be conducted, we will therefore not be able to determine whether reported outcomes are generalisable.

The results of this scoping review will be disseminated through a peer-reviewed publication and presentations at relevant conferences. In addition, the data extracted will be used for dissemination in a WHO policy brief, amongst relevant stakeholders in the international childhood cancer community.

## Supplementary Information

**Additional file 1: Supplementary file 1.** Sample database search strategy (Ovid Medline).

**Additional file 2: Supplementary file 2.** Data extraction form for Scoping Review.

## Data Availability

Not applicable

## Abbreviations

GICC: Global Initiative for Childhood Cancer
HIC: High-income countries
LIC: Low-income countries
LMIC: Low- and middle income countries
MeSH: Specific Medical Subject Headings
NGO: Non-governmental Organization
OSF: Open Science Framework
WHO: World Health Organization
