# Supplementary material for: The development and education of a workforce in childhood cancer services in low- and middle-income countries: a scoping review protocol
Source: Syst Rev. 2022 Aug 13;11:167. doi: 10.1186/s13643-022-02040-0 (PMC9375391; doi:10.1186/s13643-022-02040-0)
Supplement: Supplementary file 1 — Additional file 1: Supplementary file 1. Sample database search strategy (Ovid Medline). [file 13643_2022_2040_MOESM1_ESM.docx]

**Supplementary file 1**

Sample database search strategy (Ovid Medline)

| \|  \| [# ▲](https://ovidsp.dc1.ovid.com/ovid-b/ovidweb.cgi?&S=GALGFPMIBCACBIMEKPPJAFDKFKNKAA00&Sort+Sets=descending) \| **Searches** \| \| --- \| --- \| --- \| \|  \| 1 \| (neoplasm* or cancer* or oncolog*).tw,kf,hw. \| \|  \| 2 \| (teach* or educat* or train*).tw,kf,hw. \| \|  \| 3 \| ed.fs. \| \|  \| 4 \| (subspecialt* or specialist* or speciali#ation* or graduate* or diploma* or postgraduate* or fellowship*).tw,kf,hw. \| \|  \| 5 \| (newborn* or new-born* or baby or babies or neonat* or neo-nat* or infan* or toddler* or pre-schooler* or preschooler* or kinder or kinders or kindergarten* or kinder-aged or boy or boys or girl or girls or child or children or childhood or pediatric* or paediatric* or school-age* or schoolage* or schoolchild* or schoolgirl* or schoolboy* or adolescen* or youth or youths or teen or teens or teenage*).af. \| \|  \| 6 \| developing countries/ \| \|  \| 7 \| (austere or (limited adj2 resource*) or (low adj2 resource*) or (transitioning adj econom*) or (third adj world) or LMIC or LMICs or (lami adj countr*) or (transitional adj countr*) or (low adj gdp) or (low adj gnp) or (low adj gross adj domestic) or (low adj gross adj national) or ((emerging or developing or (low adj income) or (middle adj income) or (low adj3 middle) or underdeveloped or under-developed or (less* adj developed) or underserved or under-served or deprived or poor*) and (countr* or nation*1 or econom* or population or world))).tw,kf. \| \|  \| 8 \| exp africa/ \| \|  \| 9 \| americas/ or exp caribbean region/ or exp central america/ or latin america/ or mexico/ or exp south america/ \| \|  \| 10 \| europe/ or exp europe, eastern/ or exp transcaucasia/ \| \|  \| 11 \| antarctic regions/ or exp atlantic islands/ or exp indian ocean islands/ or exp pacific islands/ \| \|  \| 12 \| New Guinea/ \| \|  \| 13 \| asia/ or exp asia, central/ or asia, southeastern/ or borneo/ or cambodia/ or east timor/ or indonesia/ or laos/ or malaysia/ or mekong valley/ or myanmar/ or philippines/ or thailand/ or vietnam/ or asia, western/ or bangladesh/ or bhutan/ or india/ or middle east/ or afghanistan/ or iran/ or iraq/ or jordan/ or lebanon/ or oman/ or saudi arabia/ or syria/ or turkey/ or yemen/ or nepal/ or pakistan/ or sri lanka/ or far east/ or china/ or tibet/ or exp korea/ or mongolia/ \| \|  \| 14 \| (Afghanistan or Albania or Algeria or Angola or Antigua or Argentina or Armenia* or Aruba or Azerbaijan or Bahrain or Bangladesh or Barbados or Barbuda or Belarus or Byelarus* or Byelorussian or Belorussian or Belorus* or Belize or Benin or Bhutan or Bolivia or Bosnia or Botswana or Brasil or Brazil or Bulgaria or (Burkina adj Fas*) or (Upper adj Volta) or Burma or Burundi or Cambodia or Khmer or Kampuchea or Cameron* or Cameroon* or (Cape adj Verde) or (Cabo adj Verde) or (Central adj African adj Republic) or Chad or Chile or China or Colombia or Comoros or (Comoro adj Island*) or Comores or Mayotte or Congo or Kongo or (Costa adj Rica) or (Cote adj D'ivoire) or Croatia or Cuba or Czech* or Djibouti or Dominica or Dominican or (East adj Timor) or (East adj Timur) or Ecuador or Egypt or El-Salvador or (Equatorial adj Guinea) or Eritrea or Estonia or Ethiopia or Fiji or (French adj Somaliland) or Futuna or Gabon or (Gabonese adj Republic) or Gambia or Gaza or (Georgia* adj Republic) or Ghana or Grenada or Guam or Guatemala or Guinea or Guiana or Guyana or Haiti or Herzeg* or Hercegovina or Honduras or Hungary or India or Indonesia or Iran or Iraq or (Ivory adj Coast) or Jamaica or Jordan or Kazakh* or Kenya or Kiribati or Korea or Kosovo or (Kyrgyz adj Republic) or Kyrgyzstan or Kirghizia or Kirghiz or Kirgizstan or Laos or (Lao* adj2 Democratic adj Republic) or (Lao* adj PDR) or Latvia or Lebanon or Lesotho or Basutoland or Liberia or Libya or Lithuania or Macedonia or Madagascar or (Magalasy adj Republic) or Malawi or Malay* or Sabah or Sarawak or Maldives or Mali or (Marshall adj Island*) or Mauritania or Mauritius or (Agalega adj Island*) or Mexico or Micronesia or Moldov* or Mongolia or Montenegro or Morocco or Ifni or Mozambique or Myanma* or Namibia or Nauru or Nepal or (New adj Guinea) or (New adj Caledonia) or Nicaragua or Niue or Niger or Nigeria or (Northern adj Mariana adj Island*) or Nyasaland or Oman or Pakistan or Palau or Panama or (Papua adj New adj Guinea) or PNG or Palestine or Paraguay or Peru or Philipines or Philippines or Phillipines or Phillippines or Poland or (Puerto adj Rico) or Yemen or Romania or Roumania or Rumania or Russia* or Rwanda or Ruanda or (Saint adj Kitts) or (St adj Kitts) or Nevis or (Saint adj Vincent) or (St adj Vincent) or Grenadines or Samoa* or (Navigator adj Island*) or (Saint adj Lucia) or (St adj Lucia) or (Sao adj Tome) or (Saudi adj Arabia) or Senegal or Serbia or Seychelles or (Sierra adj Leone) or Slovenia or Slovak* or (South adj Africa) or (Solomon adj Island*) or Somalia or (Sri adj Lanka) or Ceylon or Sudan or Surinam* or Swaziland or Eswatini or Syria or Syrian-Arab-Republic or Tajikistan or Tadzhikistan or Tadjikistan or Tadzhik or Tanzania or Thailand or Tibet or Timor-Leste or Togo or (Togolese adj Republic) or Tokelau or Tonga or Trinidad or Tobago or Tunisia or Turkey or Turkmenistan or Turkmen or Tuvalu or Uganda or Ukraine or Uruguay or Urundi or USSR or (Soviet adj Union) or "Union of Soviet Socialist Republics" or Uzbekistan or Vanuatu or (New adj Hebrides) or Venezuela or Vietnam or (Viet adj Nam) or (United adj Arab adj Republic) or (West adj Bank) or Yemen or Yugoslavia or Zaire or Zambia or Zimbabwe or Rhodesia).tw,kf. \| \|  \| 15 \| (africa or americas or caribbean or (central adj America) or (latin adj America) or (south adj America) or (eastern adj Europe) or Transcaucasia or antarctic or (atlantic adj island*) or (indian adj ocean adj island*) or (pacific adj island*) or polynesia or (central adj asia) or (southeast* adj asia) or (south-east* adj asia) or borneo or mekong or (western adj asia) or (middle adj east) or (far adj east)).tw,kf. \| \|  \| 16 \| 6 or 7 or 8 or 9 or 10 or 11 or 12 or 13 or 14 or 15 \| \|  \| 17 \| 1 and (2 or 3) and 4 and 5 and 16 \| \|  \| 18 \| exp Workforce/ \| \|  \| 19 \| capacity building/ or health personnel/ or exp allied health personnel/ or exp health facility administrators/ or medical staff/ or exp nurses/ or exp nursing staff/ or pharmacists/ or exp physicians/ \| \|  \| 20 \| Health Planning/ \| \|  \| 21 \| personnel management/ or "personnel staffing and scheduling"/ or workload/ or personnel turnover/ or staff development/ \| \|  \| 22 \| ((human adj resources* adj2 health) or (health adj system* adj1 strengthening) or (health adj workers*) or (health adj workforce adj1 planning) or (health adj service adj1 planning) or (workforce adj1 modelling)).tw,kf,hw. \| \|  \| 23 \| 18 or 19 or 20 or 21 or 22 \| \|  \| 24 \| 1 and 5 and 16 and 23 \| \|  \| 25 \| 17 or 24 \| \|  \| 26 \| limit 25 to (case reports or guideline or practice guideline) \| \|  \| 27 \| 25 not 26 \| \|  \| 28 \| limit 27 to yr="2001 -Current" \| |
| --- | --- | --- | --- | --- | --- | --- | --- | --- | --- | --- | --- | --- | --- | --- | --- | --- | --- | --- | --- | --- | --- | --- | --- | --- | --- | --- | --- | --- | --- | --- | --- | --- | --- | --- | --- | --- | --- | --- | --- | --- | --- | --- | --- | --- | --- | --- | --- | --- | --- | --- | --- | --- | --- | --- | --- | --- | --- | --- | --- | --- | --- | --- | --- | --- | --- | --- | --- | --- | --- | --- | --- | --- | --- | --- | --- | --- | --- | --- | --- | --- | --- | --- | --- | --- | --- | --- | --- |
