# Supplementary material for: The development and education of a workforce in childhood cancer services in low- and middle-income countries: a scoping review protocol
Source: Syst Rev. 2022 Aug 13;11:167. doi: 10.1186/s13643-022-02040-0 (PMC9375391; doi:10.1186/s13643-022-02040-0)
Supplement: Supplementary file 2 — Additional file 2: Supplementary file 2. Data extraction form for Scoping Review. [file 13643_2022_2040_MOESM2_ESM.docx]

Supplementary file 2: Data extraction form for Scoping Review

| **Scoping Review details** | |
| --- | --- |
| Scoping Review Title | Workforce development and education in childhood cancer in Low-Middle Income countries (LMIC): a scoping review. |
| Review question | *What is known in the literature on the development and training of a childhood cancer workforce in low- and middle-income countries?* |
| **Inclusion/ Exclusion criteria** | |
| Population | Health professionals involved in the diagnosis and management of children with cancer across the cancer continuum |
| Concept | The development and training of a childhood cancer workforce |
| Context | Health services that diagnose, refer or treat children with cancer in low- and middle-income countries |
| Types of evidence source | Primary research studies, review articles, comments, letters, editorials, web material, policy documents, websites, country reports.  Are excluded guidelines, case reports, practice guidelines. |
| **Evidence source details and characteristics** | |
| Document/Study type (eg: original research, policy document, report…) |  |
| Citation details (eg: author, date, title, journal, volume, issue, pages) |  |
| Study design/ Methodology (eg: Qualitative, survey, review…) |  |
| Country of origin and income level (LIC, LMIC, UMIC) |  |
| Main objective of document |  |
| Context – eg childhood cancer service, mixed adult-child service, primary care |  |
| Participants or Population (details and number) – type of healthcare professional |  |
| Main action and strategy as per Framework |  |
| **Key findings** | |
| Type of challenge described or addressed |  |
| Nature of intervention |  |
| Main driver of intervention |  |
| Duration of intervention |  |
| Target healthcare professional |  |
| Type of partnership (if any) |  |
| **Key outcomes** | |
| Measure of effectiveness/ reporting of outcome used (eg improved referrals, quality of care, worker satisfaction) |  |
| Outcome of intervention |  |
| Gaps |  |
| **Other findings / suggestions for future** | |
|  | |
